# Applications of Metallic Nanoparticles in the Skin Cancer Treatment

**DOI:** 10.1155/2022/2346941

**Published:** 2022-11-14

**Authors:** Mahrokh Marzi, Mahmoud Osanloo, Mohammad Kazem Vakil, Yaser Mansoori, Abdolmajid Ghasemian, Azizallah Dehghan, Elham Zarenezhad

**Affiliations:** ^1^Noncommunicable Diseases Research Center, Fasa University of Medical Sciences, Fasa, Iran; ^2^Department of Medical Nanotechnology, School of Advanced Technologies in Medicine, Fasa University of Medical Sciences, Fasa, Iran; ^3^Department of Internal Medicine, School of Medicine, Fasa University of Medical Science, Fasa, Iran; ^4^Department of Medical Genetics, School of Medicine, Fasa University of Medical Sciences, Fasa, Iran

## Abstract

Skin cancer is one of leading cancers globally, divided into two major categories including melanoma and nonmelanoma. Skin cancer is a global concern with an increasing trend, hence novel therapies are essential. The local treatment strategies play a key role in skin cancer therapy. Nanoparticles (NPs) exert potential applications in medicine with huge advantages and have the ability to overcome common chemotherapy problems. Recently, NPs have been used in nanomedicine as promising drug delivery systems. They can enhance the solubility of poorly water-soluble drugs, improve pharmacokinetic properties, modify bioavailability, and reduce drug metabolism. The high-efficient, nontoxic, low-cost, and specific cancer therapy is a promising goal, which can be achieved by the development of nanotechnology. Metallic NPs (MNPs) can act as important platforms. MNPs development seeks to enhance the therapeutic efficiency of medicines through site specificity, prevention of multidrug resistance, and effective delivery of therapeutic factors. MNPs are used as potential arms in the case of cancer recognition, such as Magnetic Resonance Imaging (MRI) and colloidal mediators for magnetic hyperthermia of cancer. The applications of MNPs in the cancer treatment studies are mostly due to their potential to carry a large dose of drug, resulting in a high concentration of anticancer drugs at the target site. Therefore, off-target toxicity and suffering side effects caused by high concentration of the drug in other parts of the body are avoided. MNPs have been applied as drug carriers for the of improvement of skin cancer treatment and drug delivery. The development of MNPs improves the results of many cancer treatments. Different types of NPs, such as inorganic and organic NPs have been investigated *in vitro* and *in vivo* for the skin cancer therapy. MNPs advantages mostly include biodegradability, electrostatic charge, good biocompatibility, high drug payload, and low toxicity. However, the use of controlled-release systems stimulated by electromagnetic waves, temperature, pH, and light improves the accumulation in tumor tissues and improves therapeutic outcomes. This study (2019-2022) is aimed at reviewing applications of MNPs in the skin cancer therapy.

## 1. Introduction

One of the main causes of death is cancer around the world. American cancer institute has reported that the cancer accounts for the second most common cause of death after heart disease in the United States. In 2022, nine million new cancers with 609,360 deaths are projected to occur in the United States [1]. Although not being the deadliest type of cancer, the skin cancer, the unusual growth of skin cells, has an increasing trend worldwide [2], and ranks as the most common form of malignancy in developing and developed countries [3]. Skin cancer is dividing into two general groups, including melanoma and nonmelanoma. Additionally, nonmelanoma skin cancer (NMSC) is classified as basal cell carcinoma (BCC) and squamous cell carcinoma (SCC) [4].

Melanoma is the most serious and fatal type of skin cancer in spite of involving a very low proportion of skin cancers (~1%) [5, 6].

Various chemotherapeutic agents are used to treat cancer, such as antimetabolites, alkylating agents, terpenoids, and plant alkaloids. Unfortunately, major limitations have been reported for chemotherapy. Firstly, nonspecifically targeting cells (off-target toxicity) leads to a diversion of clinical outcomes due to normal cells divided quickly. Chemotherapy affects tumor cells quickly but exerts high levels of toxicity against normal cells, including the gastrointestinal tract, bone marrow, hair follicles, and macrophages [7, 8]. For this reason, medical practitioners delay their treatments with chemotherapy drugs or reduce effective doses. On the other hand, resistance to chemotherapy drugs has limited their usage [9–12]. Therefore, the development of novel anticancer drugs or alternatives for efficient treatment is crucial.

Nanotechnology is the usage of materials on an atomic, molecular, and supramolecular scale for industrial targets. The first broad description of nanotechnology refers to the specific technological goal of precise manipulation of atoms and molecules to make macroscale products, also currently known as molecular nanotechnology. Nanotechnology develops and introduces numerous novel materials and tools with a wide span of usages, like in nanoelectronics, nanomedicine, biomaterials energy production, and consumer products [13–15]. Nanotechnology is a known field of science conducted at the nanoscale presented by Nobel laureate Richard P. Feynman [16]. This has made a great revolution in the design, preparation, and fabrication of new materials in the shape and size of a nanoscale. Although the National Nanotechnology Initiative (NNI) defines nanomaterials as materials with sizes between 1 and 100 nanometers, this extent can be increased to 1000 nanometers [17]. This characteristic feature of nanostructures, due to their high surface-to-volume ratio, has increased interest in biomedical applications like treatment, diagnosis, and imaging. [18, 19]. Nanoparticles (NPs) have a wide range of applications in treating various diseases such as cancers [20, 21]. Metallic NPs (MNPs) are important agents, which offer many opportunities for biomedical usages, such as radiotherapy enhancement and diagnostic assays [22, 23]. MNPs have received much attention due to the strong electromagnetic field on the particle surface, simplicity in the synthesis method, and simple surface functionalization [24, 25]. Nanoparticle-based drug delivery systems have shown more efficient activities than those of conventional therapies. They outlined desirable pharmacokinetics, accrued targeting of tumor cells, decreased side effects, and drug resistance [26, 27]. In order to target different tumor cells, MNPs can be used in single forms or in combination with other materials such as polymers, peptides, DNA/RNA, lipids, and antibodies [28, 29]. MNPs have already gained popularity in targeted drug delivery system through active and passive targeting (by performing tumor imaging). Suitable functional groups give properties to these particles [30].

This research is focused on the latest papers on the application of MNPs (2019-2022) in the treatment of skin cancer.

## 2. Methods

**Figure 1:**
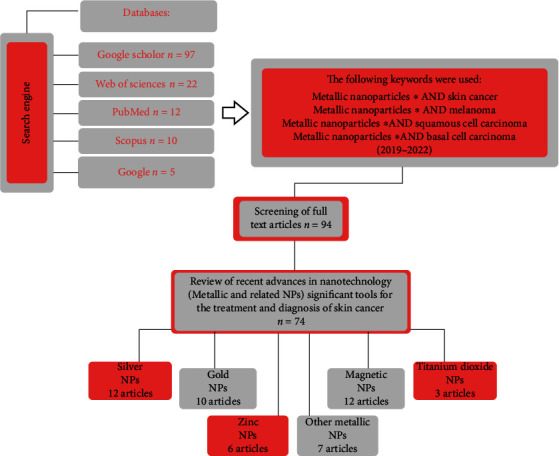
Content charts provided in this review.

Recent advances in nanotechnology (metal and related NPs) are significant tools for treating and recognizing skin cancer, as discussed in this study. The literature search is performed on the Web of Science, PubMed, Google, Scopus, and the Google Scholar databases. The following keywords were used: Metallic NPs ^∗^AND skin cancer, Metallic NPs ^∗^AND melanoma, Metallic NPs ^∗^AND squamous cell carcinoma, and Metallic NPs ^∗^AND basal cell carcinoma. (Figure 1).

### 2.1. Selection Criteria

In this paper, the inclusion criteria included were as follows: (1) the relevant papers which accessed the nanocarriers as a promising tool for efficient treatment of skin cancer, (2) observational study, English published, and available full texts. Additionally, the exclusion criteria included were as follows: (1) expert opinions, (2) conference abstract, case series and case report, (3) editorials, and (4) other cancers instead of skin cancer.

### 2.2. Study Screening

The EndNote version X.9.1 was used for those publications, which met our criteria. All duplicates were removed. Full text manuscript was used when the abstract or titles had no enough information.

## 3. Nanocarriers as a Promising Instrument for Efficient Treatment of Skin Cancer

### 3.1. Nanoparticles

**Figure 2:**
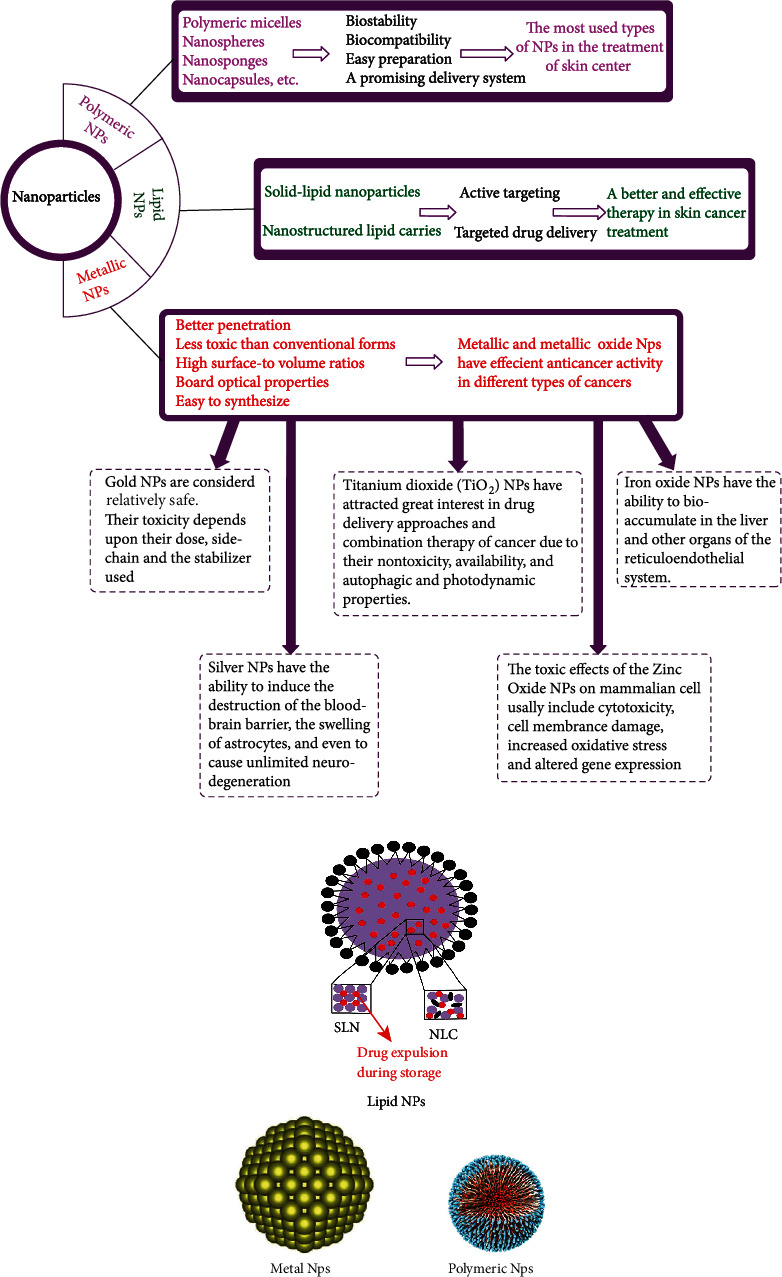
Nanoparticles as a promising tool for the efficient treatment of skin cancer. (a) Classification of types of nanoparticles. (b) Shape of nanoparticles [34–36].

NPs are composed of particles dispersed below nanometers or solid particles with a size span of 10-1000 nm with biomimetic attributes [31, 32]. Biomimetic attributes increase in combination with the high surface-to-volume proportion. The capability to improve these properties in biomedical usage increases with potential applications in imaging, diagnosis, and therapy [33] (Figures 2(a) and 2(b)).

#### 3.1.1. Polymeric Nanoparticles

Polymer NPs are composed of one or more polymers with various molecular weights, hydrophobic chains, and structures. These NPs are available in polymer micelles, nanospheres, nanosponges, nanocapsules, and so on [33, 37, 38]. Polymeric micelles are nucleated shell micelle complexes consisting of more than one polymer chain with different hydrophobicity [39–41]. *N*-isopropylacrylamide/vinyl pyrrolidone heat-resistant nanopolymer micelles were loaded with paclitaxel and appraised for their anticancer property in skin melanoma cell line (B16F10) and breast cancer cell line (MCF-7). The nanopolymer system had therapeutic efficacy with a stable release effect and displayed biocompatible compatible attributes [42]. Targeted folic acid micelles from synperonic PE/F 127-cholesteryl hemisuccinate created by Varshosaz et al. [43] through dialysis, in which docetaxel was included for the efficient therapy of melanoma. According to the obtained results, micelles had more cytotoxic effects with higher cell uptake and effective reduction of tumor volume. Polymeric nanomicelles are one of the most effective carriers of chemotherapeutics for the selection of skin cancer cells and have impressive cytotoxic efficacies.

Nanospheres are spherical polymer NPs, in which therapeutic compounds are evenly distributed all over the polymer matrix. Nanospheres have a high trapping yield, small size span, flat surface, and controlled and stable release model. Nanospheres are also effective in treating skin cancer. Surface changes in nanospheres may increase the effectiveness of skin cancer treatment because they modify the level of toxicity and increase cell uptake and cytotoxicity [44]. Nanosponges are NPs with nanometer lattice structures and nanometer-wide pores and could enclose lipophilic and hydrophilic materials. Nanosponges increase the solubility of insoluble materials. They are insoluble, porous, nontoxic in water and organic solvents, and stable at temperatures up to 300°C (compared to other NPs) [45]. Nanosponges could carry water-insoluble medicine and are composed of drugs and polymers. Nanocapsules are nanovesicular systems, in which the drug can be placed in liquid or solid shape or the form of a molecular dispersion in a reservoir or cavity surrounded by a polymeric membrane or coating and identified by the structures of the nucleus shell [46]. Various studies have proven the effectiveness of nanocapsules containing therapeutic agents in treating skin cancer [47–49].

#### 3.1.2. Lipid Nanoparticles

Lipid NPs were evaluated as drug delivery systems in the early nineteenth century by R. H. Müller and M. Gascon. These NPs were composed of solid lipids or a mixture of liquid and solid lipids by emulsifiers as stabilizing factors [50, 51].

*(1) Solid-Lipid Nanoparticles*. SLNs due to their skill in combining hydrophilic and lipophilic drugs, better physical and chemical stability for sensitive molecules, lower skin irritation, potential for drug delivery to specific and controlled locations, particle size reduction, high and effective drug load, low-cost production, avoidance of carrier toxicity, etc. are known as alternative drug delivery systems for polymer nanoparticles, liposomes, and emulsions [52, 53].

*(2) Nanostructured Lipid Carriers*. As a nanocarrier system (nanostructured lipid carriers), it can allow drugs to penetrate deeper substrates of the skin and provide a better location for topical delivery of effective parts. Through the use of NLCs, the skin absorption of drugs can be significantly increased, which may be due to their distinctive combination of spatially incompatible dual lipids (lipids and solid oils), which has provided a higher encapsulation efficiency for a combination drug [54, 55]. Nanostructured lipid carriers (NLCs) are made from a mixture of solid and liquid lipids without proper crystal structure. The lipid is either surrounded by the solid lipid matrix or on the surfactant layer [56]. Therefore, the liquid phase with reduced water amount provides high medicine loading and the solid lipid fraction provides properties for controlled drug release [57]. Also, increasing the distance between the fatty acid chains and the unstructured crystal supports the increased drug loading in NLCs. Therefore, they are much more suitable for drug formulation compared to SLN [58].

#### 3.1.3. Metallic Nanoparticles

MNPs have a diversity of biomedical usages, including their use in sensitive detection, drug delivery, genetic material, thermal abrasion, and radiation therapy enhancement [22, 59–61]. MNPs have better permeability and less toxicity than noninfected cells compared to conventional forms. The important role of MNPs in the clinical context of cancer treatment is extensive optical properties, high surface-to-volume ratio, easy synthesis and chemistry, and facial surface function [59, 62, 63]. MNPs and metal oxide NPs (MONPs) have effective anticancer activity in various types of cancer, such as breast cancer [64], lung [65], colorectal [66], pancreatic [67], and skin cancers [68]. Different MNPs and MONPs have a better cytotoxic efficacy on skin cancer than conventional dosage forms. MNPs are several times more effective than simple drugs *in vitro*. Nirmala et al. [69] developed gold/Au NPs stabilized with Vitis vinifera skin polyphenols to obtain effective cytotoxic effects and induce apoptosis by increasing reactive oxygen species (ROS) production and significantly reducing membrane potential in the A431 skin cancer cell line. Other MNPs and MONPs applications include detecting and imaging cancer cells [70], making them promising for treating and assessing skin cancer.

**Figure 3:**
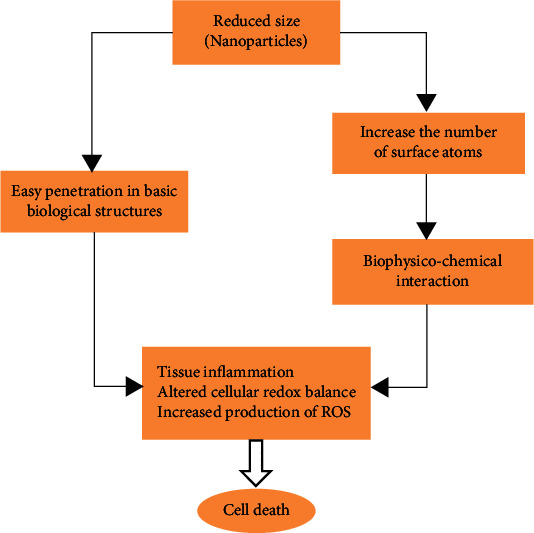
Toxicity of metallic nanoparticles, ROS: reactive oxygen species.

*(1) The Toxicity and Disadvantage of Metallic Nanoparticles*. MNPs are useful in various fields; however, some concerns remains regarding their adverse effects on human health and the environment [71]. Direct and indirect disposal of MNPs in the environment also causes toxic effects. The nanosize of MNPs adds to the number of surface area and the surface atoms causing biophysicochemical interaction of MNPs with the physiological environment [72]. Also, their small size helps to interpenetrate in the biological structures [73]. The interaction of MNPs with cellular proteins and enzymes leads to the production of reactive oxygen species (ROS) [74]. This further leads to mitochondria dysfunction, which is associated with an increased inflammatory response. This destruction of mitochondria causes apoptosis [75] (Figure 3).

For the treatment of skin cancer, for example the BCC, various types of NPs such as MNPs, polymeric NPs, and lipid NPs have been proposed to load a wide range of chemically differentiated anticancer medicines [76, 77].

## 4. Nanotechnology (Metallic and Related NPs) Are Significant Tools for the Treatment and Diagnosis of Skin Cancer

The main characteristics of MNPs include high surface-to-volume ratio, high surface energies, transition between molecular and metallic states, short-range ordering, and large number of low coordination sites. In attention to these properties that exist at the nanometer scale, MNPs are gaining a lot of interest and expansion [78].

The skin is an obstacle protecting the body against environmental allergens, chemicals, and harmful substances. This distinctive barrier comprises the epidermis, dermis, hypodermis, and many appendages such as hair follicles, sweat glands, and sebaceous glands. The ability of MNPs to overcome this skin barrier and penetrate the deep layers of the skin is controversial. The interaction of NPs with the skin is yet to be fully understood, as it has been shown that some NPs can penetrate the outer layer of the stratum corneum, while others can penetrate deeper into the skin layer and enter the systemic circulation [79]. Numerous factors affect MNPs skin adsorption, such as shape, charge, size, formation, and surface modification. The MNPs effect on the skin cancer cell lines has been exhibited in Table 1.

NPs: nanoparticles; ROS: reactive oxygen species.

The biggest impact on the prognosis of any type of skin cancer in early detection and immediate treatment. Although various types of nanoparticles have been shown to affect the growth of melanoma cells in vitro and in vivo, only a few types of nanomotors have been approved for clinical trials [87] (Table 2).

### 4.1. Ag Nanoparticles (AgNPs)

Shivashankarappa and Sanjay [80] synthesized Ag NPs by the bacterial strain *Bacillus licheniformis* and used photodynamic therapy (PDT) of 5-aminolevulinic acid (5-ALA) conjugated with synthesized Ag NPs (microbial) on epidermoid carcinoma (A431) and skin melanoma (B16F10) cell lines. The cytotoxicity of NPs coupled with 5-aminolevulinic acid and pure NPs was evaluated by polyethylene glycol (PEG) on cell lines (B16F10 and A431) and compared with the 5- aminolevulinic acid standard. This study showed that 5-ALA could be effectively combined with microbially synthesized Ag NPs via electrostatic interaction. The conjugated 5-ALA AgNPs demonstrated superior cytotoxicity in both cell lines than each single forms of AgNPs and the pure ALA and produced higher levels of the ROS molecules due to the formation of protoporphyrin IX (PpIX) in cells when exposed to radiation. Photodynamic therapy can be successfully used to treat cancer by combining NPs with photosensitizer medicine molecules. The antimicrobial properties of AgNPs were performed on food-borne pathogens, and the NPs revealed antimicrobial activity comparable to reference antibiotics.

A systematic measure to appraise the antiproliferative and antitumor results of AgNPs in melanoma under standard conditions was performed according to the National Institute of Health (NIH) suggested protocol. These NPs were covered with polyvinylpyrrolidone (PVP). The average size of NPs was 35 ± 15 nm and a Ag-metal amount of 1.2% wt. Major variations in cell livability, infusion of apoptosis and necrosis, and ROS production were discovered in B16-F10 cells after 6 hours of exposure to Ag particles or cisplatin (IC_50_ = 4.2 *μ*g/ml and IC_50_ = 2.0 *μ*g/ml). The better biocompatibility of these AgNPs than cisplatin was revealed as the infusion of apoptosis, mostly in the mitochondria (the major route of cell death after six hours of ROS overproduction). *In vivo* experiments also displayed which therapies of 3, 6, and 12 mg/kg AgNPs produced a survival rate of about 4 times higher than cisplatin. In addition, AgNPs-treated surviving mice did not demonstrate the genotoxic harm determined by quantifying micronucleus frequency in surrounding blood cells. These findings demonstrate the significant antitumor effect of a nontoxic AgNPs formulation and the first progress towards using these AgNPs to treat melanoma, which can significantly alleviate the damaging effects of current chemotherapies [94].

Capanema et al. [95] designed and developed a new dual-purpose hybrid hydrogel for use against skin cancer cells with antimicrobial activity, comprising AgNPs inserted in carboxymethylcellulose (CMC) polymer matrices attached to citric acid and the combination with doxorubicin (DOX). These hybrids were developed using an all-green chemistry strategy for nanomedical usage against skin cancer. Supermolecular nanostructures were fabricated using CMC polymers (in situ chemical reduction of Ag^+^ by a single-pot). These nanostructures (AgNPs@CMC) were effectively combined with doxorubicin using the electrostatic interplays that make up colloidal nanocomposites. To produce hybrid hydrogel networks with modified properties, these nanoconjugates were bonded with citric acid using the CMC carboxylation surface (degree of substitution (DS) = 0.8 and 1.2). The findings showed impressive nucleation and stability of spherical nanocolloids of AgNPs with identical size dispensation (d ~10 nm). In addition, nanoconjugates (AgNP @CMC-DOX) were mostly created using electrostatic interplays between cationic amino groups (DOXs) and anionic carboxylate groups (CMCs) that formed complexes with vesicle-similar nanostructures diffused in water. These AgNP@CMC-DOX nanostructures with gel diminution and swelling degree attributes dependent on the degree of substitution were created to produce hybrid hydrogels (AgNP@CMC-DOX-CA). As a result, these hybrids showed a synergistic efficacy of Ag particles in killing skin cancer cells due to the DOX-regulated intracellular kinetics *in vitro*. In general, topical drug delivery chemotherapy can use this new hybrid hydrogel platform containing nanoparticle-polysaccharide-medicine joined systems as a novel weapon against skin cancer.

In this research, a hydrogel film comprising bovine serum albumin (BSA) was coated with AgNPs, and its application was appraised for topical photothermal treatment (PTT) of skin cancer. The synthesized BSA/AgNPs showed a small size dispensation with good size strength with an excellent photothermal activity that could be sustained by repeated laser irradiation. According to these experiments, when testing for anticancer results in B16F10 s.c. tumor-carrying mice, PTT with topical therapy of hydrogel films loaded with BSA/AgNP can especially prevent tumor growth with treatment without apparent toxicity [96].

Kim et al. [97] developed the BSA-covered AgNPs and identified multifaceted therapeutic activities of NPs *in vitro* for the therapy of skin cancer. BSA-AgNPs, provided by the single-stage reduction method, revealed good resistance and suitable size dispensation for pharmaceutical appeals. These NPs demonstrated specific cytocidal results on melanoma cells, possibly due to oxidative stress. BSA-AgNPs induced considerable antiangiogenic effects on human umbilical vein endothelial cell (HUVEC) via preventing multiplication, immigration, and tube establishment. In addition, BSA-AgNPs showed the ability to convert light into significant heat, indicating their use as photothermal factors.

This study is aimed at developing AgNPs on carboxylate nanocrystal cellulose (cCNC) to minimize and reduce the number of chemical reagents with constant anchor and controlled release of Ag ^+^ using hydrothermal reaction. Ag-carboxylate nanocrystal cellulose was effectively created using ammonium persulfate (APS) oxidized cellulose and hydrothermal reaction in two stages to minimize the chemical reagent as reducing or stabilizing ammonium persulfate for surface-modified and Ag nitrate for the hydrothermal reaction. Ag^+^-controlled emission was also assessed, and evaluated the anticancer activity of SK-MEL-2 (Human Skin Melanoma cells). AgNPs were evenly sedimented on carboxylate nanocrystal cellulose with an efficiency of 28% and a particle size of about 15 nm. The release speed of Ag ions for 32 days was assessed using ICOES analysis. Ag-carboxylate nanocrystal cellulose (Ag-cCNC) was found to release Ag ions slowly. Ag-cCNC displayed a 15% higher anticancer effect than cisplatin but was 18% lower in anticancer SK-MEL-2. This work presents a new, stable, easy, and simple environmentally friendly method for producing and using natural composite substances from Eucalyptus as an antitumor medicine [98].

An effective and inexpensive biodegradable treatment was initiated with AgNPs, chitosan, and sericin compositions by Nayak et al. [99]. Then, different ethosomal formulations were appraised as a substrate for the dermal delivery vehicle for effective skin interposition therapies. In the first place, the ethosomes operate as a carrier that assists the encapsulated medicine in attaining its target site. Then, AgNPs release free radicals that disrupt the homeostatic balance within cancer cells and destroy their mitochondria and DNA machines. The formulations use all new, natural, and cost-effective resources that work synergistically to provide an alternative approach to targeting the nonmelanoma skin carcinoma (NMSC) with the least chance of medicine resistance and cancer relapse. An identical mixture of AgNPs and sericin facilitated the morphological and cellular metamorphosis of epidermoid A431 skin cancer cells. The *in vivo* results showed the stimulation of immunoglobulins (IgM) discharge with T cell-mediated protected response. Finally, using biocompatible formulas presented through ethosomes, this research suggests a new approach to the treatment of NMSC.

Hamdi et al. [88] appraised the anticancer activity of green synthesized AgNPs of *Gallium aparine* extract. According to the tests performed, the diameters range of AgNPs was from 35-110 nm. Various concentrations (6.225-400 *μ*g/ml) of petroleum ether extract and its green AgNPs were examined as antiskin cancer. Findings show that *G. aparine* extract is a desirable biological bearer for synthesizing AgNPs that can be used in various fields of biomedicine and pharmaceutical. The synthesis method of NPs is easy, environmentally friendly, and effective in developing various multifunctional NPs that can be practical in environmental and nanomedical usages.

Kuang et al. [92] generated small-sized AgNPs covered with sucrose (S-AgNPs) as powerful assistants in finding a successful combination treatment strategy. The antitumor effects of AgNPs coated with sucrose were investigated and partially evaluated in immunodeficient and immunocompetent mice. Fluorescence-activated cells were isolated, and immunofluorescence painting analysis was performed to recognize tumor microenvironments. According to the experiments, S-AgNPs showed strong antitumor results, tiny systemic toxicity, and favorable druggability. In this study, it was discovered that S-AgNPs displayed higher antitumor effects in mice with good immunity. Furthermore, this research has shown that S-AgNPs repress tumor cell reproduction using inducing cell apoptosis and increasing the penetration and activity of cytotoxic CD8 + T cells (Mechanically). Clinically, S-AgNPs have shown high positional antitumor activity and moderate systemic safety with PD-1 mAbs in inhibiting melanoma multiplication, which offers a new combined clinical therapy strategy.

Himalini et al. [93] have reported an approach with environmentally-friendly and cost-effective by easy procedures for the NPs biosynthesis of several fungal species of *Fusarium* spp. Fungi discharge amounts of enzymes and proteins that operate as decreasing and masking agents in synthesizing AgNPs. AgNPs were produced by the extracellular fungi extract of *Fusarium incarnatum*. The antitumor activity of fungal extracts, AgNPs, and reference drug (kojic acid) against SKMEL-3 melanoma cells of human skin was investigated. The medium size of AgNPs was 10 nanometers and was mostly spherical. Mycosynthesis of AgNPs revealed considerable antimelanogenic activity as opposed to human skin melanoma SK-MEL-3 cells (IC_50_ value of 17.70 *μ*g/ml). The concentration of AgNPs necessary to prevent SK-MEL-3 cells was less than the concentrations of *F. incarnatum* extract and reference drug. A promising candidate for study in the pharmaceutical industry is the result of efficient antimicrobial activity and remarkable antimelanogenic activity of NPs synthesized on human skin melanoma cells in a very small concentrations.

In a study, AgNPs were prepared using a variety of antioxidant compounds containing (^_^)-epicatechin-3-gallate (EGCG), caffeine (CAF), and gallic acid (GA). Due to physicochemical properties, each type of AgNPs showed a spherical form, negative surface charge, and comparable size dispensation. The chemistry of AgNP-stabilizing shells is an important factor in regulating the toxicity of AgNPs against human skin melanoma cells and murine B16 melanoma cells (according to surface-enhanced Raman spectroscopy). (^_^)-epicatechin-3-gallate AgNPs (EGCGAgNPs) had the highest cytotoxicity among all AgNPs studied. They extremely decreased the mitochondria activity, harmed cell membrane entirety, interpenetrated inside the cells, and damaged DNA. The toxicity of gallic acid AgNPs (GAAgNPs) was revealed through the infusion of oxidative stress in cells. CAFGAAgNPs showed lowest toxicity against melanoma cells and demonstrated that the proper combination of antioxidants could prepare AgNPs with distinct toxicity. Human skin melanoma cells were found to be remarkably more sensitive to AgNPs than mouse melanoma cells [90].

Rahim et al. [100] investigated the biogenic synthesis of *Alstonia angustiloba* -AgNPs by the aqueous extract of *A. angustiloba* leaves and evaluated its antiproliferative mechanisms. The cytotoxicity of *A. angustiloba* aqueous extracts and *A. angustiloba*-AgNPs were evaluated using the MTT experiment towards the A431 cancer cell line. The NPs displayed a spherical form (average size of 61.21 ± 3.96 nm) and a zeta potential value (–18.67 ± 3.12 mV). After 72 hours of treatment, the NPs inhibited the growth of A431 cells (IC 50 value of 39.58 *μ*g/ml). The NPs also induced apoptosis and cell cycle arrest in A431 cells. Consequently, *A. angustiloba* biosynthesized AgNPs could be utilized for potential anticancer usage.

### 4.2. Gold Nanoparticles

A strong and cost-effective biocompatible gold/AuNP was synthesized using an aqueous *Siberian ginseng* extract by Wu et al. [81]. According to the physicochemical traits, the synthesized AuNP met the entire characters of a strong AuNP. The results demonstrated a surface plasmon resonance peak at 538 nm with stability until 30 days of incubation outlining the spherical form, size of the synthesized AuNPs, and crystalline substance. These findings showed that the biological parts in Siberian ginseng reduce gold ions and synthesize NPs. After identification, the efficacy of Siberian ginseng-AuNPs opposite to melanoma was evaluated *in vitro* by melanoma cells of B16 mice. SG-GNPs increased the ROS levels and reduced mitochondrial membrane potential. Therefore, the use of SG-GNPS reduced the antiapoptotic proteins and enhanced the rate of proapoptotic proteins in melanoma cells (acts as a mimic of trihydridoboron (BH_3_)).

The use of different conjugates of AuNPs in treating skin cancer in mice has been investigated. The simpler process of synthesizing and combining AuNPs with doxorubicin (Dox), nisin, and nisin-doxorubicin was achieved with a potential control of size and shape, colloidal resistance, and adjustable surface chemistry. It was speculated that nisin interacts with the membrane of cancer cells and leads to the formation of pores. This may increase the uptake of nisin-GNPs/nisin-Dox-GNPs into cancer cells. Thus, Dox/AuNPs were liberated in larger values into intracellular cancer cells and inhibited DNA synthesis or other intracellular efficacies. Their results significantly displayed the modified anticancer potential of AuNPs, nisin-, Dox-, and nisin-Dox-conjugated AuNPs against skin cancer *in vivo* [101].

In another study, the induction and mechanism of elective apoptosis in melanoma G361 cells were investigated by AuNPs conjugated to anti-HER2 antibody (cancer marker). Increased BAX (a protein coding gene) was detected in cells undergoing apoptosis, caspase-3 and -9 activation, and poly (ADP-ribose) polymerase and DNA fragmentation agent 45 (caspase-activated DNase inhibitor) in GNP-HER2 treatment. After GNP-HER2 treatment, an increase in cells was perceived in the sub-G1 phase, a signal of cellular apoptosis. Then GNP-HER2 therapy was approved to induce cell cycle advancement to cease. Reductions in phospho-focal coherence kinase or phospho-human epidermal growth agent receiver and reductions in phospho-paxillin were detected to activate cellular focal coherence and stimulate the separation of filamentary actin, respectively. Decreased cell adhesion and separation of intracellular construction indicate cell inactivation. In general, GNP-HER2 selectively destroys G361 melanoma cells without influencing normal cells [102].

Pesnel et al. [103] investigated the plasmonic thermal degradation of mice melanoma using AuNPs acquired by green chemistry. The data were attained using measurement of tumor content and mice weight in various classes of B16F10 mouse melanoma treated or not with the NPs and laser irradiation. The data were examined to compare the antitumor efficacy of photothermal plasmonic treatment by AuNPs and the standard therapy. In case of at least 20% weight loss of the mouse (according to the criteria of the National Cancer Institute), a toxic dose was considered.

Li et al. [104] prepared the AuNPs and perfluorinated hexane and conjugated melanoma-related antigens (MAGE) targeting melanoma with monoclonal antibody (MAGE-1 antibody), evaluated the synergistic result of PTT/ODV (photothermal therapy/optical droplet vaporization) treatment examined by ultrasound. By combining the effects of PTT and ODV, complete tumor irradiation was obtained being controlled by contrast ultrasound. Several benefits included noninvasiveness, short recovery time, low side effects, and controlled treatment procedure. In addition, the prepared MAGE-Au-PFH-NPs eliminated tumors. These new targeted NPs can be acted as a multifunction theranostic factor for the tumor-guided imaging.

A research is aimed at investigating the effects of low and true Au- and AgNPs concentrations on melanoma of B16F1 and B16F10 mice, making these cells more invasive and metastatic. No toxicity was found against the B16 cells by neutral red, trypan blue, crystal violet, and MTT assays at 0.01 to 10 ng/ml concentrations. Partial toxicity for B16F10 exposed at 100 ng/ml was observed with a decrease in the number of viable and connected cells, indicating different sensitivities of B16F1 and B16F10 cells to NPs. In addition, colony size dispersal was reduced for both B16 cell lines. Both NPs are widely used in nanotechnology products, and no documented assayed concentrations of Au- and AgNPs can make B16 cells more aggressive and malignant [83].

Chi et al. [105] studied whether 5-aminolevulinic acid- (5-ALA-) conjugated GNPs could increase the antitumor performance of photodynamic therapy (PDT) in CSCC and investigated the principle molecular mechanisms. The results revealed that PDT with 5-aminolevulinic acid and AuNPs-conjugated 5-ALA (5-ALA-GNPs) seriously repressed cell life, enhanced cell apoptosis, and singlet oxygen production in HaCat and A431 cells, and photodynamic therapy with 5-ALA and 5-ALA-GNPs in A431 cells was more effective than HaCat cells. In addition, treatment with 5-ALA-GNPs compared with 5-ALA treatment enhanced the effects of PDT on cell viability, cell apoptosis, and single oxygen production in A431 cells. Further experiments displayed that PDT with 5-ALA-GNPs reduced STAT3 and Bcl-2 expression and developed Bax expression in A431 cells compared with PDT with 5-ALA.

This study is aimed at inspecting the effect of various sizes of AuNPs combined with different ultrasound intensities on the survival of melanoma cancer cells. In addition, the size efficiency of AuNPs on acoustic cavitation was explored. For this purpose, ultrasonic irradiation was applied with intensities of 0.5, 1, and 2 watts per square centimeter and a frequency of 1 MHz in the presence of AuNPs with cysteamine-folic acid conjugate (F-Cys-GNP) with sizes of 15, 23, and 79 nm and different concentrations (0.2, 1, and 5 *μ*g/ml). This was done at various incubation times of 12, 24, and 36 hours. Melanoma cell viability is reduced at higher sizes and concentrations of F-Cys-GNPs. The lowest melanoma cell viability was observed in cells containing 79, 23, and 15 nm F-Cys-GNPs. Therefore, nucleation sites at the GNPs level enlarge with increasing GNPs size, which leads to an enhancement in the number of cavitation bubbles [106].

Rajendran et al. [107] utilized ferulic acid (fa), a polyphenol, to increase the AuNPs stability. Thus, it operated as a reducing factor when combined with hydrogen tetrachloroaurate (III) hydrate at environment temperature. Ferulic acid became a stabilizing factor and produced spherical AuNPs (fa-AuNPs). After identification, the synthesized fa-AuNPs were inquired in normal keratinocytes (HaCaT cells) and human skin cancer cells (A431). The fa-AuNPs induced cytotoxicity in A431 cells in a dose- and time-dependent way. CAM assay results demonstrated the angiogenic effect of fa-AuNPs. The programmed cell death happened through apoptosis which was displayed using the sub-G1 population. Mitochondrial membrane potential decreased with increasing the level of the ROS and caspase-3 activity.

In a survey, a combination of chemotherapy and ultrasound treatment in the form of sonodynamic therapy (SDT) with the presentation of AuSiO_2_ NPs conjugated with dacarbazine (DTIC@AuSiO_2_) NPs as a sonosensitizer for the treatment of melanoma. The sonosensitization activity of AuSiO_2_ NPs and higher absorption of dacarbazine by tumor cells after loading in DTIC@AuSiO_2_ NPs effectively prevented the proliferation of melanoma tumor cells. As a result, the DTIC@AuSiO_2_ NPs were useful delivery medium and sonosensitizer platform for use in the sonodynamic treatment of melanoma [108].

### 4.3. Zinc Oxide Nanoparticles (ZnO-NPs)

The present work seeks to investigate the cytotoxic capacity of zinc oxide NPs (ZnO-NPs) in addition to the human melanoma cell line (A375). Cell viability was resolved, and ZnO-NPs demonstrated potential cytotoxicity. Adhesion and cell morphology were determined using the propidium iodide method. The mRNA expression of apoptotic genes such as caspases 3, 8, and 9 increased following exposure to ZnO-NPs. It was revealed ZnO-NPs stimulate apoptotic cell necrosis at the transcriptional step. *Cardiospermum halicacabum* adjusted the apoptotic gene expressions. Accumulation of ROS increased in a concentration-dependent manner, which normalized multiple index pathways and manipulated cellular kinetic actions. Synthesized ZnO-NPs of *C. halicacabum* may persuade cell necrosis through high ROS levels in cells. CH-ZnONPs further stimulate apoptotic markers and exacerbate cancer cell necrosis, cell toxicity, and increased ROS. In general, mixed CH-ZnONPs of *C. halicacabum* had significant toxicity to human melanoma cells (A375) by stimulating apoptosis and necrosis, which required the practical efficacy of CH-ZnO-NPs in addition to malignant management [109].

The toxicity of ZnO-NPs using mouse melanoma cell line (B16) was reported at different concentrations on melanoma cells. Considering the ability of ZnO-NPs to inhibit cancer cell proliferation in rat embryonic fibroblast cell line, it was concluded that an inhibitory activity for the growth of these cancer cells was statistically significant (very significant). The findings displayed that ZnO-NPs were sensitive to (morphological) changes in the number, shape, and size of linear B16 cancer cells for melanoma, which were monoclonal cells [85]. It confirms that ZnO-NP is safe and efficient candidates for cancer cell growth restriction. Thus, ZnO-NPs are expected to be considered as a novel class of anticancer factors.

do Reis et al. [110] developed a drug delivery system containing dacarbazine and light sensitizing factor (zinc phthalocyanine) by MV3 melanoma cells as a pattern. The dual emulsion method generated polylactic acid/polyvinyl alcohol (PLA/PVA) NPs containing dacarbazine and zinc phthalocyanine. The NPs had an average diameter of ~259 nm with a spherical form. Therapeutic photodynamic experiments *in vitro* indicated that the compound was critical and dose-dependent for increasing therapeutic efficacy. *In vitro* cytotoxicity assays using endothelial cells showed that the drug encapsulated in the NPs had no considerable toxicity compared to reference samples. The results showed that drug loading influences the biological distribution of NPs formulations. The findings showed that the offered NP formulation could be used to treat the photodynamics of melanoma. Biodistribution/tissue deposition experiments showed that the mononuclear phagocytic system adsorbs most of NPs formulations, not recommended for the treatment of melanoma (as a systemic application). However, topical application was the suitable route for delivery. The data confirmed that dual encapsulated NPs conferred better *in vitro* effects than each compound singly.

In a study, ZnO-NPs were synthesized from the rhizome extract of *Alpinia calcarata,* and their biotherapeutic attributes were determined. ZnO-NPs reveal a better biocidal activity against *E. coli* and *C. albicans* than other microorganisms. Furthermore, ZnONP treatment showed a dose-dependent decrease in human epidermoid carcinoma A431 cell viability by MTT assay. In addition, it marked morphological changes associated with apoptosis by acridine orange/ethidium bromide (AO/EB) staining [111].

Czyżowska et al. [112] defined the mechanism of primary toxicity of ZnO-NPs to cells based on the interaction with the lipid portion of the native cell membrane and the model. The chosen cell lines behave differently in contact with NPs. In this study, abnormalities in the native membrane of B16-F0 cells and, to a lesser extent, in COLO 679 were observed. The membrane of COLO 679 cells was more peroxidated, and cell livability was much shorter. Due to the obtained physicochemical parameters for single and composite lipids, it was concluded that exposure to NPs lead to changes in model membranes (especially in the polar parts of lipids). The highest interaction was observed between ZnO-NPs and zwitterionic phospholipids (PC and PE), cholesterol, and phosphatidylglycerol with a negative charge.

This study is aimed at evaluating the effect of ZnO-NPs on melanocytes under situations associated with a high degree of epidermal barrier dysfunction to determine whether ZnO-NPs can cause malignant melanocyte transformation after penetration of damaged skin. ZnO-NPs increased oxidative damage, inhibited apoptosis, and increased expression of nuclear factor kappa B (NF-*κ*B) p65 and Bcl-2 in skin melanocytes with epidermal barrier dysfunction after continuous treatment for 14 and 49 days. Exposure to 5.0 *μ*g/ml ZnO-NPs for 72 hours increased cell viability, decreased apoptosis, and increased Fkbp51 expression in melanocytes, according to histological observations *in vivo*. Ongoing exposure to ZnO-NPs has an antiapoptotic effect on melanocytes by activating NF-*κ*B pathways through oxidative stress, both *in vitro* and *in vivo*. Due to the risk of melanoma associated with repeated and continuous exposure to ZnO-NPs in conditions of epidermal barrier dysfunction, this study provided new information. [113].

### 4.4. Magnetic Nanoparticles (MNPs)

Duval et al. [82] used a quantitative RNA, nanostringing, and western blot protein analyses technique to evaluate the protein and gene expression of B16 melanoma mouse cells following medium magnetic NPs (MNPs) hyperthermia (mNPH) dose equivalent to 30 minutes @43°C and or a clinically related to the radiation dose of 8 Gy. Melanoma cells were pelleted with MNPs and subjected to an alternating magnetic field (AMF) to produce the desired thermal dose (2.5 *μ*g Fe/106 cells). The mNPH dose showed a significant increase in the HSP70 gene tolerating heat/immunogenicity and some receptor gene pathways of adsorbent chemistry and toll-like receptors. The 8 Gy dose also increased the number of important genetic and protein pathways for immunity and cytotoxicity. This study shows that low-dose mNPH and radiation alone significantly increase the expression of immune and cytotoxic genes, but this effect is greatly enhanced when used in combination.

A study is aimed at exploring the inhibitory efficacy of chitosan MaNPs (CMaNPs) loaded with angiopoietin-2 (Ang-2) small intervening RNA (Ang2-siRNA) plasmids (Ang2-CMaNPs) on virulent melanoma. Melanoma progression was remarkably inhibited using Ang2-CMaNP treatment. Ang2-CMaNP treatment impressively prevented tumor growth and in situ Ang-2 expression compared with the reference group. In addition, Ang2-CMaNP treatment inhibited tumor angiogenesis and enhanced cell apoptosis by regulating the Bax/Bcl-2 ratio and increasing the expression of cleaved caspase-3 *in vivo*. The apoptosis rate of malignant melanoma cells in the Ang2-CMaNP group was notably higher than the control group (*P* < 0.05). The levels of Bcl-2 were decreased after treatment with Ang2-CMaNPs. The Bax/Bcl-2 ratio was significantly higher in the tumor tissue of the Ang2-CMaNP group than in the control group (*P* < 0.05). In general, Ang2-CMaNP treatment enhanced normal vascular regression in the TME and induced melanoma cells through the mitochondrial apoptosis pathway [114].

A survey demonstrated the accessibility of the chorioallantoic membrane (CAM) model as an instrument to experiment with the therapeutic attributes of novel nanocomposites synthesized on external melanoma grafts. This work explores the effects of salicylic acid-activated iron oxide NPs (SaMNPs) as a form of therapy on the local development of external ligaments and CAM vessels by a chicken CAM model implanted with xenografts extracted from C57BL/6 mice with B16F10 melanoma cells. This slowed the xenograft transplant volume and reduced the ability of melanoma cells to localize and distally metastasize. In addition, the use of the chick CAM model was displayed as a tool to test the performance of newly synthesized nanocomposites on external melanoma grafts. SaMNPs had a therapeutic effect on melanoma B16F10 due to the synergistic effect of its two components, nucleus of iron oxides with cytotoxic effect and coating of salicylic acid with antiangiogenic and chemotherapeutic effect [84].

Pandesh et al. [115] synthesized and reported Fe_3_O_4_@Au core-shell NPs, magnetically aimed them against a tumor, and utilized them for photothermal treatment of cancer. The efficient diameter of Fe_3_O_4_@Au NPs was nearly 37.8 nm. The mean tumor volume in the reference group, laser irradiation group, NPs group, NPs + laser irradiation group, and NPs + magnet + laser irradiation group enhanced 47.3, 45.3, 32.8, 19.9, and 7.7 times 2 weeks, respectively. There was no apparent change in mean body weight for the various groups. The results showed that this report's nanophotothermal treatment of magnetic target cancer holds great promise for selective tumor destruction.

Mîndrilă et al. [116] used a syngeneic B16F10 melanoma model implanted in C57BL/6 mice to appraise phenotypic variations in melanoma induced by treatment with salicylic acid-functionalized iron oxide NPs (SaIONs). This research displayed that oral administration of aqueous dispersion of SaIONs was pursued by a phenotypic change to high pigment cells in melanoma B16F10 by a cytotoxicity-induced cell election mechanism. In addition, hyperpigmentation of melanoma cells using intracellular or extracellular agglomeration of melanic pigment residues was another therapeutic outcome of SaIONs.

Zhang et al. [117] expanded a transdermal nanoplatform (+)T-SiDs, based on superparamagnetic iron oxide core, surface-modified with cationic lipids, transdermal increased peptide TD, and 1,1′-dioctadecyl 3,3,3′,3′-tetramethylindotricarbocyanine iodide (DiR), and loaded with doxorubicin. Compounds (+) T-SiDs enable dual magnetic resonance/near-infrared (MR/NIR) imaging, which guides synergistic chemotherapeutic-photothermal therapy to treat superficial tumors through dermal delivery. The (+) T-SiDs show good resistance, effective cellular absorption, pH/photothermal responsive medicine diffusion, and high photothermal transformation yield (47.45%). The transdermal delivery of (+) T-SiDs is notably increased using TD performance. The (+) T-SiDs show a high skin function and specificity at tumor site localization (according to MR/NIR imaging *in vivo*). In addition, a combination of photothermal chemotherapy has more effective results on tumor control than individual chemotherapy or photothermal treatments. This study offers a novel transdermal nanoparticle for photothermal-chemotherapy imaging with dual conduction of exterior tumors, with effective tumor eradication and low systemic toxicity, therefore offering the powerful potential for clinical acceptance.

This work explored the efficacy of hsa-miR-203a-3p overexpression on the multiplication of human cutaneous squamous cell carcinoma (CSCC) cells and its possible mechanism. In this research, a lentivirus vector was applied to transfer cells to improve our understanding of nanogenic transport technology and enable the potential future use of nanogenic transport technology in the treatment of CSCC. A luciferase reporting system was constructed to confirm the targeted regulatory relationship of miR-203a-3p with APC, and a miR-203a-3p lentivirus overexpression vector was constructed and used to translocate SCC-1 CSCC cells. MiR-203a-3p displayed declined expression in skin squamous cell carcinoma (SCL-1) cells and CSCC examples. The results showed that the ratio of Renilla luciferase to Firefly luciferase was reduced in SCL-1 cells of the APC 3′-UTR + miR-203a-3p group than those of the APC 3′-UTR + negative control group. After lentiviral putrefaction of SCL-1 cells, the affluence of miR-203a-3p and phosphorylated protein-catenin increased, while the abundance of APC and -catenin decreased significantly. Cell phenotyping analysis exhibited that miR-203a-3p reduced cell multiplication. MiR-203a-3p prevents SCL-1 cell proliferation by targeted regulation of APC and may act as a tumor suppressor gene [118].

This study is aimed at inspecting the potential of using stiffness-based criteria that show the inherent biophysical changes of *in vivo* melanoma tumors after hyperthermia treatment of MNPs (using the tranostatic function of MNPs). Significant changes in mechanical resonance frequency were observed only in the MH-treated group (magnetic nanoparticle hyperthermia), indicating a change in heat-induced stiffness in the melanoma cells. In addition, tumor cellularity, protein composition, and temperature increase contribute to tumor stiffness changes after MH treatment. The tumor softens after MH by low cell volume even with increasing low temperature. This paper utilized the tranostatic function of MNPs and examined the MH-induced stiffness change in vivo mice with magnetomotive optical coherence tomography (MM-OCT) and magnetomotive optical coherence elastography (MM-OCE) for the first time. It was found that the elastic change of melanoma tumors after MH treatment depends on the tumor's heat dose and morphological characteristics. Changes in elasticity at the tissue surface could potentially be a physically and physiologically significant indicator of physically and physiologically integrated treatment for MH, while MM-OCE could be a suitable dosimetric method [119].

This study is aimed at evaluating the efficacy in the photodynamic treatment of iron oxide NPs (*γ*-Fe_2_O_3_ NPs), synthesized by laser pyrolysis method, functionalized with 5, 10, 15, 20- (Tetra-4-sulfonatophenyl) porphyrin tetraammonium (TPPS), on melanoma cells of human skin, after only 1 minute of exposure to blue light. Single oxygen efficiency was assessed as the standard for porphyrin, and Rose Bengal functionalized NPs through transient properties of single oxygen phosphorescence at 1270 nm. Biological tests on the anti-cancer effect of the *γ*-Fe_2_O_3_-NPs_TPPS complexes deciphered inhibiting tumor cell proliferation, reducing cell adhesion, and inducing cell death through ROS produced by TPPS exposed to light. Predicted toxicity experiments exhibited the compound potential safety, needing further testing. In addition, the low binding energy between pro-caspase-3 and TPPS indicated a possible tendency of TPPS to inactivate caspase [120].

Li et al. [121] has investigated the effective delivery of chlorin e6 using polyglycerol-coated iron oxide NPs with conjugated doxorubicin for advanced photodynamic treatment of melanoma. Iron Oxide NPs (IO-NPs) were coated with polyglycerol (PG) for good water solubility. Doxorubicin (DOX) chemotherapy factor was attached to the PG coating through a hydrazone bond to induce a hybrid affinity for the cell membrane, thereby enhancing cell uptake. The hydrophobic nature of DOX also led to the accumulation of IO-NPs to form nanoclusters. Chlorin e6 was then loaded onto IO nanoclusters by physical adsorption and coordination with surface iron atoms to obtain the final IO-PG-DOX-Ce6 composites. The results showed that IO-PG-DOX-Ce6 significantly increased the uptake of Ce6 in rat melanoma cells, leading to greatly increased photocytotoxicity, enhanced ROS levels, lost viability, DNA damage, and stimulation of tumor cell immunogenesis were identified. *In vivo* tests confirmed *in vitro* findings and showed long-term blood purification of IO-PG-DOX-Ce6. IO-PG-DOX-Ce6 significantly enhanced Ce6 dispensation and retention in rat subcutaneous melanoma grafts and significantly boosted the yield of Ce6-mediated photodynamic therapy (PDT). No apparent damage to vital organs was observed simultaneously. As a result, IO-PG-DOX NPs provide an easy and secure delivery platform for efficient Ce6 tumor enrichment, thereby enhancing antimelanoma PDT.

A study is aimed at expanding new multilayer nanofilms containing a composition of polyhydroxyethyl methacrylate (PHEMA), polyhydroxypropyl methacrylate (PHPMA), sodium deoxycholate (NaDOC) with incorporated superparamagnetic iron–platinum NPs (FePt NPs), and the strong anticancer medicine (5-fluorouracil), for the treatment of theranostic skin cancer. The drug release activity was tested *in vitro,* and the formulation's safety was appraised on human skin-derived fibroblasts. The prepared combination revealed the capability of controlled drug release to prevent undesirable side effects, which is a higher attribute of novel drug delivery systems. In addition, the multilayer structure enabled easy yet effective dose changes and represents a promising personal therapeutic approach. This study demonstrated the useful therapeutic effect against the BCC and immunosuppression of basal matrices on human-derived skin fibroblasts [122].

In a study, magnetite (Fe_3_O_4_) NPs were synthesized by simultaneous deposition and dispersed through an emulsion polymerization process in a poly (vinyl pivalate) thermoplastic matrix. Improving the magnetic response of polymeric magnetic materials using polymerizable carboxylic acids as coating agents and minimizing the leaching of NPs during nanocomposite formation was the main goal of separate encapsulation of MNPs. Thus, the magnetite surface synthesized by acrylic acid or methacrylic acid was modified to improve its individual encapsulation during the polymerization stage, thus producing a series of magnetic nanocomposites comprising various amounts of magnetite intended for biomedical usages. The results showed an average size of approximately 8 nm for pure MaNPs and spherical morphology. Fe_3_O_4_ had an acidic agent size of approximately 6 nm, while the size of the nanocomposites was approximately 7 nm. Magnetization evaluation supplied a saturation magnetization amount of almost 75 emu/g and vouched for a superparamagnetic manner at room temperature. Experiments with homopolymers and magnetic composites against different cell lines (e.g., fibroblasts, keratinocytes, and human melanoma) to evaluate cytotoxicity levels outlined acceptable results at different times and concentrations of exposure, resulting in more than 70% cell survival compared to the control group [123].

### 4.5. Titanium Dioxide Nanoparticles

A study is aimed at mechanically appraising the potential of titanium dioxide (TiO_2_) NPs to increase the effects of chemotherapy *in vitro* and *in vivo* models of rat melanoma. The F10 melanoma cells were subjected to various concentrations of TiO2 NPs and/or cisplatin, then cell growth, cell livability, and cell death were assessed. Nontoxic concentrations of TiO_2_ NPs (50 *μ*g/ml) increase the antiproliferative and cytotoxic effects of cisplatin on F10 melanoma cells by induction of autophagy and necrotic cell death. While TiO_2_ NPs exerted no cytotoxic or metastatic effects on mice melanoma cells, their combination with cisplatin increased the drug response (up to 50%) and led to higher antitumor effects compared to each monotherapy. The combination of TiO_2_ NPs with cisplatin increased the response to chemotherapy in *in vitro* and *in vivo* melanoma models. In addition, autophagy played a key role in the sensitization of melanoma cells to chemotherapy [86].

Another study described the synthesis of quercetin-conjugated TiO_2_ nanotubes (TNT–Qu) and exhibited the favorable effect of TNT–Qu on anticancer activity *in vitro* against B16F10 melanoma cells. TNT-Qu treatment prevented migration compared to single TNT (14.14%) or quercetin (44.86%) treatment and remarkably induced 60.29% apoptosis in melanoma cells. In addition, quercetin and TNT-Qu reduced ROS and superoxide levels due to the antioxidant properties of quercetin. The molecular mechanism of the TNT–Qu on melanoma cells increased the caspase-3 levels and provoked the apoptosis more potentially than that of each TNT or quercetin singly. Thus, the novel TNT-Qu conferred higher anticancer effects, considered as a promising skin cancer combination therapy [124].

Gulla et al. [125] explored the efficacy of quercetin, TNT, or the TNT–Qu in the B16F10 mouse melanoma model and two-step chemical carcinogenesis model by 7,12-Dimethylbenz (a) anthracene as a tumor primer, and phorbol 12-myristate-13 acetate as a tumor promoter. The topical application of TNT-Qu increased antitumor activity compared to single forms of each quercetin or TNT. TNT-Qu prevented tumor growth by means of regulation of phospho-STAT3 in the TME without any effects on the skin color and deferred the fatality rate in tumor-bearing mice. Histopathological results demonstrated that the TNT-Qu treatment reduced the CSCC compared to each of quercetin or TNT singly and substantially improved the epidermal hyperplasia. TNT-Qu treatment reinstated the of *γδ*T cells to the surface of normal skin. In addition, TNT-Qu prevented the formation of blood vessels in chick chorioallantoic membrane tests.

### 4.6. Other Metallic Nanoparticles (Palladium, Nickel Oxide, Platinum, Selenium, Copper, Vanadium, and Cerium Oxide)

Tris (dibenzylideneacetone) dipalladium (Tris DBA-Pd), with and without antibodies to IGF1R, was successfully loaded on the hyaluronic acid NPs. Tris DBA-Pd inhibited the *N*- myristoyltransferase 1 (NMT-1) with activity against melanoma cells *in vivo*. In this study, Tris DBA-Pd hyaluronic acid NPs (Tris DBA-Pd HANP) synthesized and assessed against xenografts of LM36R, an aggressive BRAF mutant human melanoma persistent to BRAF inhibitors (to boost its therapeutic effect and prevail drug resistance in advanced melanomas). Xenografted mice have been treated in four forms including HANPs, free Tris DBA-Pd, Tris DBA-Pd HANPs, and Tris DBA-Pd HANPs plus IGF1R antibody. The Tris DBA-Pd HANP group showed the highest response to treatment and the highest decrease in CD44 positive cells in the immunohistochemistry (IHC). Amazingly, the HANPs containing IGF1R antibody, were less efficient than particles without antibody (due to steric inhibition of IGF1R and CD44 binding). Tris DBA-Pd NPs are a useful treatment for CD44-positive tumors like melanoma [126].

This work evaluated the cytotoxic effects of nickel oxide NPs (NiO-NPs) on melanoma mitochondria. The results showed that NiO-NPs increased the levels of the ROS, mitochondrial membrane potential, and lipid peroxidation. Besides, NiO-NPs cause lysosomal membrane labilization in the mitochondria. NiO-NPs could notably induce optional cytotoxicity on malignant cutaneous melanoma (MCM) mitochondria. Therefore, this compound can be considered a favorable candidate for further *in vivo* and clinical studies to achieve a novel antimalignant melanoma medicine [127].

Platinum NPs (PtNPs) efficacy was appraised as a sensitizer for photothermal treatment (PTT), radiotherapy (RT), and composed PTT and RT (PTT/RT) in a mouse malignant melanoma cell line (B16/F10). Two laser power densities (1.0 and 1.5 W cm^−2^) and three X-ray doses (2, 4, and 6 Gy) were selected for B16/F10 cell line irradiation 24 and 72 hours after treatment. The synthesized PtNPs had a spherical form with a 12.2 ± 0.7 nm diameter and were cytocompatible up to 250 *μ*g ml^−1^. A concentration-dependent photothermal conversion activity was observed at 72 h after treatment. PtNPs showed cytotoxicity on X-ray doses of 2, 4, and 6 Gy after 24 hours, while 72 hours later resulted in deeper consequences. Dual radiation of laser light and X-ray to PtNPs considerably improved the treatment by producing the ROS. PtNPs can operate as a new dual absorber of laser light and X-ray, a usual sensitizer used to treat cancer [128].

Mohammadi et al. [129] have explored laser and ultrasound irradiation efficacies using selenium-polyethylene glycol-curcumin (Se-PEG-Cur) NPs on melanoma cancer. Selenium-polyethylene glycol-curcumin was recommended as a new factor for absorbing 808 nm laser light and ultrasound (US) for the treatment of C540 (B16/F10) cancer cells. Then, ROS production in C540 (B16/F10) cancer cells on PTT and SDT was measured using Se-PEG-Cur. Cell survival after laser irradiation or US waves at 100 *μ*g/ml Se-PEG-Cur decreased (33.9% and 22.9%, respectively). Detection of intracellular ROS showed that dual PTT and SDT in Se-PEG-Cur attendance induced the greatest ROS production. Thus, Se-PEG-Cur was introduced as an agent for absorbing laser light and US waves to treat cancer.

Zhang et al. [130] studied whether the antitumor efficacy of copper-cysteamine- (Cu–Cy-) based photodynamic therapy (PDT) was comforted using an enhancement in the influence of immune cells containing DCs, M1 macrophages, CD4^+^T cells, and NK cells in tumors. Flow cytometric investigation revealed that DCs, CD8^+^T cells, and NK cells had the highest ratio in Cu–Cy + X-ray-treated tumor tissues. Overall, the results showed that Cu-Cy-mediated PDT could induce strong antitumor immune responses through DC maturation. In summary, after X-ray activation, Cu-Cy NPs can produce significant levels of ROS, leading to direct melanoma destruction. In addition to producing ROS, Cu-Cy NPs along with X-rays can impressively convince an antitumor immune response. In general, Cu-Cy NPs can activate radiotherapy, oxidative therapy, and immunotherapy simultaneously for cancer treatment and help prevail the restrictions of traditional cancer treatment methods.

An easy method was developed for synthesizing irregular dumbbell vanadium pentoxide NPs (NPs V2O5: 30-60 nm) through irradiating polyhedral microwaves with calcination. The cell viability experiments revealed that V_2_O_5_ NPs could prevent the multiplication of various cancer cells (B16F10, A549, and PANC1) that show their antiproliferative activity. Also, V_2_O_5_ NPs did not exhibit considerable cytotoxicity to the normal cells (CHO, HEK-293, and NRK-49F), indicating their biocompatible essence. Furthermore, these NPs prevented the multiplication and immigration of the endothelial cells (HUVECs and EA.hy926) and disturbed the blood vasculature in chick embryo patterns, indicating their antiangiogenic attributes. Mechanical research showed that efficient internalization of NPs V_2_O_5_ produces intracellular ROS, which regulate p53 protein and survivin protein in cancer cells, leading to apoptosis. In addition, administration of V_2_O_5_ NPs to mice with C57BL6/J melanoma raised their survival compared to uncontrolled mice and demonstrated the therapeutic potential of NPs against melanoma. Further, the *in vivo* toxicity experiment showed no toxic results in mice after chronic exposure to V_2_O_5_ NPs [131].

Yong et al. [132] have investigated ROS-the mediated antiangiogenic activity of cerium oxide NPs in melanoma cells. Due to the ability of cerium oxide NPs (nanocrystals) to modulate intracellular ROS, nanomaterials are favorable for biomedical usage. This study investigated the result of temperature, precipitating factor concentration, add speed, stirring rate, and surfactant concentration on the nanoparticle particle scale using a fractional factorial experimental plan method. At doses up to 400 *μ*g/ml, these NPs are not cytotoxic to a human melanoma cell line (Mel1007) and are dose-dependently internalized via the cells. As a result, intracellular ROS levels decrease for some cells that internalize the nanoceria, which is associated with a dose-dependent decrease in the expression of angiogenic genes containing antivascular endothelial growth factor (VEGF).

## 5. Conclusions

The high-efficient, nontoxic, low-cost, and specific cancer therapy is a promising goal, which can be achieved by the development of nanotechnology. MNPs have been applied as drug carriers for skin cancer drug delivery. The development of MNPs improves the results of many cancer treatments. Different types of NPs, such as inorganic and organic NPs, have been used *in vitro* and *in vivo* treatment of skin cancer types. MNPs were considered due to their great biodegradability, electrostatic charge, good biocompatibility, high drug payload, and low toxicity. By using these particles, diagnosis and treatment can be improved simultaneously, and drug delivery can be implemented in a controlled and targeted manner, which was one of the main drawbacks of the conventional drug delivery system. Researchers have also made progress in using these NPs as a diagnostic tool for imaging cancer cells in the early stages, and the main reason for this is the gene silencing and better gene targeting activities of these particles. However, the use of controlled-release systems stimulated by electromagnetic waves, temperature, pH, and light, improves the accumulation in tumor tissues and enhances therapeutic outcomes.

## Figures and Tables

**Table 1:** An overview of related studies included herein.

Year	Type of particles	Cells	Results	Ref.
2019	Silver nanoparticles	B16F10 and A431 cell lines	ROS production in 5-ALA silver nanoparticle conjugates in both B16F10 and A431 cells was highest in irradiated cells, indicating drug activation	[80]
2019	Gold nanoparticles	Murine B16 melanoma cells	The CC50 dose of Siberian ginseng gold NPs against B16 murine melanoma cells was very low	[81]
2019	Magnetic nanoparticle	B16-F10 murine melanoma cells	Magnetic NPs hyperthermia and low-dose radiation independently increase the expression of important immune and cytotoxic genes, enhanced in combination therapy	[82]
2020	Silver and gold nanoparticles	Murine melanoma B16F1 and B16F10 cells	No confirmation of AuNPs and AgNPs effects on B16 cells aggressiveness and malignancy	[83]
2020	Iron oxide/salicylic acid nanoparticles	Murine B16 melanoma cells	Iron oxide NPs functionalized with salicylic acid had therapeutic potential in B16F10 melanoma (salicylic acid coating with antiangiogenic and chemotherapy function and iron oxide core with cytotoxic effect)	[84]
2021	Zinc oxide nanoparticles	Murine B16 melanoma cells	Exerted significance inhibitory activity against the growth of cancer cells	[85]
2021	Titanium dioxide (TiO2) nanoparticles	F10 melanoma mouse cell line	TiO_2_ nanoparticles have no cytotoxic or metastatic effects in melanoma mice, its combination with cisplatin increases the drug response (up to 50%), and leads to greater inhibition of tumor growth compared to either monotherapy	[86]

**Table 2:** Several types of metal nanoparticles proven for clinical trials.

Year	Type of particles	Cells	Results	Ref.
2021	Silver nanoparticles	Human malignant melanoma (A375 cell line, ATCC number CRL-1619)	The nanoparticles have shown good anticancer activity towards human skin cancer cells	[88]
2021	*A.calamus*-zinc oxide nanoparticle (AC-ZnONPs) coated cotton fabrics	Human skin melanoma SK-MEL-3 cells	AC-ZnONPs efficiently suppressed the viability of SK-MEL-3 cells	[89]
2022	Silver nanoparticles	Human and murine skin melanoma cells	Human skin melanoma cells were found to be significantly more sensitive to AgNP than mouse melanoma cells	[90]
2022	Cerium oxide nanoparticles	Human melanoma cell line, Me1007	The produced nanoceria was successfully internalized without conferring cytotoxicity to melanoma cells	[91]
2022	Silver nanoparticles	Human metastatic melanoma cell lines A375 and SK-MEL-28 and the murine melanoma cell line B16-F10	The newly obtained silver NPs coated with sucrose had better solubility and stability in water, with antitumor capacity inhibiting melanoma proliferation and enhanced antitumor immunity	[92]
2022	Silver nanoparticles	Human skin melanoma SK-MEL-3 cells	Biosynthesis of spherical NPs from *F. incarnatum* fungal extract showed antimicrobial and antimelanogenic activity of synthesized silver NPs on SK-MEL-3 human skin melanoma cells	[93]

## Data Availability

The datasets used and analyzed during the current study are available from the corresponding author on reasonable request. We have presented all data in the form of figures.

## Abbreviations

5-ALA: 5-aminolevulinic acid
A431: Epidermoid carcinoma cell lines
ADP-ribose: Adenosine diphosphate ribose
Ag-cCNC: Ag-carboxylate nanocrystal cellulose
AMF: Alternating magnetic field
Ang-2: Angiopoietin-2
Ang2-siRNA: Ang-2 small interfering RNA
AO/EB: Acridine orange/ethidium bromide
APC: Is a member of the Wnt signal transduction pathway, in which APC and *ß*-catenin form a complex resulting in *ß*-catenin degradation
APS: Ammonium persulfate
Au-NPs: Gold nanoparticles
BAX: BCL2 associated X, apoptosis regulator is a protein coding gene
B16F10: Skin melanoma cell lines
BH_3_: Trihydridoboron
BSA: Bovine serum albumin
CA: Citric acid
CAF: Caffeine
CAM assay: Chorioallantoic membrane
cCNC: Carboxylate nanocrystal cellulose
CEM43 30: Immunogenetic effects of low dose
CH: Cardiospermum halicacabum
CMC: Carboxymethylcellulose
CMNPs: Chitosan magnetic nanoparticles
CSCC: Cutaneous squamous cell carcinoma
Cu–Cy: Copper-cysteamine
DiR: 1,1′-dioctadecyl-3,3,3′,3′-tetramethylindotricarbocyanine iodide
DNA: Deoxyribonucleic acid
DOX: Doxorubicin
DS: Degree of substitution
DTIC@AuSiO_2_: AuSiO_2_ core-shell nanoparticles conjugated with dacarbazine
EGCG: (^_^)-epicatechin-3-gallate
EGCGAgNPs: (^_^)-epicatechin-3-gallate Ag nanoparticles
fa: Ferulic acid
F-Cys-GNPs: Gold nanoparticles with cysteamine-folic acid conjugate
FePt NPs: Iron–platinum nanoparticles
GA: Gallic acid
GAAgNPs: Gallic acid Ag nanoparticles
GNPs: Gold nanoparticles
HaCaT: Normal keratinocytes
HANP: Hyaluronic acid nanoparticles
HER2: A cancer marker
HSP70: Protein expression
HUVEC: Human umbilical vein endothelial cell
IC50: Half-maximal inhibitory concentration
IGF-IR (3B7): Monoclonal antibody: Is recommended for detection of IGF-I
IgM: Immunoglobulins
IHC: Immunohistochemistry
IO-NPs: Iron oxide nanoparticles
mAbs: Monoclonal antibodies
MCM: Malignant cutaneous melanoma
MH: Magnetic nanoparticle hyperthermia
miRNAs: microRNAs
MM-OCT: Magnetomotive optical coherence tomography
mNPH: Magnetic nanoparticle hyperthermia
mNPs: Magnetic nanoparticles
MONPs: Metal oxide nanoparticles
MRI: Magnetic resonance imaging
mRNA: Messenger ribonucleic acid
MR/NIR: Magnetic resonance/near infrared
MTT assay: A colorimetric assay for measuring cell metabolic activity (3-(4,5-dimethylthiazol-2-yl)-2,5-diphenyltetrazolium bromide)
MV3: Melanoma cell line
NaDOC: Sodium deoxycholate
NF-*κ*B: Nuclear factor kappa B
NIH: National Institute of Health
NiO-NPs: Nickel oxide nanoparticles
NLCs: Nanostructured lipid carriers
NMSC: Non melanoma skin carcinoma
NMT-1: *N*- myristoyltransferase 1
NNI: National Nanotechnology Initiative
NO: Nitric oxide
NP: Nanoparticle
O^2-^: Superoxide
-OH: Hydroxyl
Pd: Palladium
PD-L1: Programmed cell death protein 1 ligand
PDT: Photodynamic therapy
PEG: Polyethylene glycol
PFH: Perfluorohexane
PG: Polyglycerol
PHEMA: Polyhydroxyethyl methacrylate
PLA/PVA: Poly lactic acid/polyvinyl alcohol
PpIX: Photoporphyrin IX
PtNPs: Platinum nanoparticles
PTT: Photothermal therapy
PTT/ODV: Photothermal therapy/optical droplet vaporization
PVP: Polyvinylpyrrolidone
ROS: Reactive oxygen species
RT: Radiotherapy
S-AgNPs: Ag nanoparticles covered with sucrose
SaIONs: Iron oxide nanoparticles functionalized with salicylic acid
SaMNPs: Salicylic acid-activated iron oxide nanoparticles
SC: Stratum corneum
SCL-1: Skin squamous cell carcinoma
SDT: Sonodynamic therapy
Se- PEG-Cur: Selenium-polyethylene glycol-curcumin
SG-GNPs: Siberian ginseng-gold nanoparticles
SK-MEL-2: Human skin melanoma cells
SLNs: Solid-lipid nanoparticles
TD: Transdermal enhanced peptide
The Bcl-2 family: Including Bcl-2, Bax, Bak, etc., is the protein group that controls the stimulation and suppression of natural cellular death and is localized in the mitochondrial inner membrane, cytoplasm, and endoplasmic reticulum
TiO_2_: Titanium dioxide
TNT: Titanium dioxide nanotubes
TNT–Qu: Quercetin-conjugated titanium dioxide nanotubes
TPPS: 5,10,15,20- (tetra-4-sulfonatophenyl) porphyrin tetraammonium
Tris DBA-Pd: Tris (dibenzylideneacetone) dipalladium
Tris DBA-Pd HANP: Tris DBA-Pd hyaluronic acid nanoparticles
(+) T-SiDs: Transdermal nanoplatform
US: Ultrasound
VEGF: Antivascular endothelial growth factor
ZnO: Zinc oxide
ZnONPs: Zinc oxide nanoparticles.
